# A Randomized Trial Evaluating the Synergistic Effect of Parenteral Diclofenac and Paracetamol for Pain Management in Adult Males With Acute Limb Injury

**DOI:** 10.1155/prm/5587917

**Published:** 2026-01-31

**Authors:** Isma Qureshi, Raheel S. Qureshi, Tim Harris, Sameer A. Pathan, Ashraf M. El Malik, Mohd E. Babiker, Mohamad A. Jamali, Zain A. Bhutta, Stephen H. Thomas

**Affiliations:** ^1^ Department of Emergency Medicine, Hamad Medical Corporation, Doha, Qatar, hamad.qa; ^2^ Department of Emergency Medicine, Blizard Institute of Barts & the London School of Medicine, Queen Mary University of London, London, UK, qmul.ac.uk; ^3^ The Alfred Hospital, Emergency and Trauma Centre, School of Public Health and Preventive Medicine, Monash University, Melbourne, Australia, monash.ac.za; ^4^ Department of Pharmacy, Cleveland Clinic, Cleveland, Ohio, USA, clevelandclinic.org; ^5^ Department of Emergency Medicine and Services, Helsinki University Hospital and University of Helsinki, Helsinki, Finland, helsinki.fi; ^6^ Department of Emergency Medicine, Beth Israel Deaconess Medical Center and Harvard Medical School, Boston, Massachusetts, USA, harvard.edu

## Abstract

**Introduction:**

In the emergency department (ED), commonly used analgesics for pain management are nonsteroidal anti‐inflammatory drugs (NSAIDs), paracetamol, and opioids. The aim of this clinical trial was to evaluate the effectiveness of intravenous (IV) or oral (PO) paracetamol, combined with intramuscular (IM) diclofenac, in patients with acute limb injuries.

**Methods:**

This study utilized a double‐blind, randomized controlled design to evaluate three different treatment groups. The trial included healthy adult males aged 18–65 years, who arrived at the ED with acute limb injuries, and an initial pain score of at least 5 on the Numerical Rating Scale (NRS). Participants were randomly assigned in equal numbers to one of three groups: one group received IM diclofenac (75 mg/3 mL), along with oral paracetamol (1000 mg); the second group was given IM diclofenac plus IV paracetamol (1000 mg in 100 mL); and the third group received IM diclofenac (75 mg/3 mL), accompanied by a placebo. The main goal of the study was to compare the average pain reduction among the three groups at 30 min after treatment (t30).

**Results:**

A total of 162 participants were recruited between October 2022 and February 2023. Pain levels were assessed at baseline (t0) and continued to be monitored up to 90 min after medication was administered (t90). The average reduction in pain scores for each group was as follows: diclofenac plus oral paracetamol resulted in a mean decrease of 2.5 ± 0.03, diclofenac plus IV paracetamol had a mean reduction of 2.6 ± 0.03, and diclofenac with placebo showed a mean decrease of 2.2 ± 0.04. These results indicate there was no statistically significant difference in pain relief among the three groups. Additionally, none of the groups required rescue pain medication, and no adverse events were reported in any group.

**Conclusion:**

The results demonstrate that the three treatment groups achieved similar levels of pain relief within the observed timeframe, offering no significant advantage in terms of speed or extent of pain reduction. Nonetheless, additional studies are warranted to explore the potential synergistic effects of combining paracetamol with NSAIDs via various administration routes, as well as to assess possible adverse events, and the necessity for supplemental analgesia.

**Trial Registration:** ClinicalTrials.gov identifier: NCT04199572

## 1. Introduction

Pain is a frequent reason for patients to visit the emergency department (ED), occurring in more than two‐thirds of ED cases [1, 2]. Trauma and limb injuries are commonly managed in the ED, resulting from a variety of mechanisms, including falls, vehicle collisions, sports, and work‐related accidents [3, 4]. Prompt and effective administration of analgesia is highly valued by both patients and healthcare providers, with pain assessment considered a “vital sign” alongside other physiological signs [5].

In the ED, commonly administered pain medications include nonsteroidal anti‐inflammatory drugs (NSAIDs), opioids, and paracetamol [6]. NSAIDs work by inhibiting prostaglandin synthesis through a range of peripheral and central actions. Paracetamol primarily provides pain relief by activating central descending serotonergic pathways [7, 8]. Paracetamol is widely utilized in intravenous (IV), rectal, and oral forms and is considered to have a favorable safety profile. NSAIDs are also frequently prescribed and can be given orally (PO), intramuscularly (IM), or IV; however, they are linked to several adverse effects, such as gastrointestinal bleeding and renal impairment, particularly when used over extended periods [9]. Opioids are often chosen for the management of moderate‐to‐severe pain, but they are associated with a host of side effects, including respiratory depression, nausea and vomiting, constipation, and addiction [10]. Research indicates that paracetamol can reduce the need for narcotics [11], whether used alone or alongside other medications, in various clinical settings such as postoperative pain [12, 13], cancer‐related pain [14], and in combination with regional anesthesia [15].

There is a scarcity of evidence on the use of multimodal analgesia in the ED [16]. Data are limited regarding both the synergistic effects of combining paracetamol and NSAIDs, and the comparative effectiveness of IV versus oral paracetamol [17]. Therefore, the authors conducted a randomized, double‐blind, controlled trial [18] to evaluate the effectiveness of IV or oral paracetamol in combination with diclofenac, or diclofenac alone, for managing pain in patients with acute limb injuries.

## 2. Methods

### 2.1. Setting and Study Participants

A prospective, double‐blind, randomized controlled trial was conducted at an urban ED, which serves an annual patient population of approximately 450,000 and is situated in an industrial area. The study focused on adult male patients aged 18–65 years, presenting with isolated acute limb injuries and a Numerical Rating Scale (NRS) pain score of 5 or higher. Screening was carried out by a dedicated research team between 07:00 and 23:00, 7 days a week. The NRS is an 11‐point scale and is routinely used in EDs for both clinical care and research purposes [19], ranging from 0 (no pain) to 10 (worst imaginable pain). Patients were excluded if they had received any analgesia within 8 hours prior to assessment, or injuries typically necessitating opioids as per the physician’s clinical judgment, such as certain severe fractures or dislocations, and were hemodynamically unstable following trauma. Additionally, participants with known allergies to either diclofenac or paracetamol, or with any relative or absolute contraindication to the study drugs, NSAIDs, and paracetamol, including stroke, bronchial asthma, gastrointestinal bleeding, renal impairment, or asthma, were excluded.

Informed written consent was obtained in each participant’s preferred language, in accordance with local guidelines and the Declaration of Helsinki. The trial received approval from the Ethics Committee of the Institutional Review Board at the Medical Research Center, Hamad Medical Corporation, Qatar (Approval number: IRGC‐04‐NI‐17‐099).

### 2.2. Intervention, Randomization, and Masking

Eligible participants were randomly allocated in equal proportions (1:1:1) to receive one of the following: IM diclofenac with oral paracetamol, IM diclofenac with IV paracetamol, or IM diclofenac with placebo. A research coordinator, who was not involved in drug preparation or data collection, created a computer‐generated randomization list using blocks of three, six, and nine. Each day, a clinical pharmacist assembled the study drug packs based on this random list. The active medications and placebos were indistinguishable in appearance and were labeled with preassigned codes. The clinicians, research assistants, participants, and statisticians were all blinded to the treatment allocations, in accordance with the double‐blind study design.

### 2.3. Procedure

Each trial pack included three components: a syringe for IM administration, a 100‐mL IV fluid bag, and two oral (PO) tablets. All trial packs were prepared under strict aseptic conditions. Each pack was labeled with a sticker displaying a unique code, the preparation date, the expiration date, and instructions for administration. To ensure bioequivalence, the study drugs were sourced from the same manufacturers. The contents of each trial pack were as follows:

• Syringe A: 3 mL solution containing either 75‐mg diclofenac or a placebo (normal saline),
• Mini‐bag B: 1‐g paracetamol in 100 mL solution or a placebo (normal saline), and
• Tablet C: two 500‐mg paracetamol tablets (totaling 1 g) or a placebo (sugar tablets) (see Table 1).

No additional medications were administered to participants during the first 30 min of the study. After 30 min, the treating physicians reassessed the patients and, as needed, provided rescue analgesia other than the drugs used in the trial (NSAIDs or paracetamol). Pain scores were recorded at the following time points: t0, t15, t30, t45, t60, t75, and t90 min. Adverse events were monitored throughout the 90‐min study period. To further evaluate adverse events, a survey was conducted by questioning the ED physicians during routine care after 90 min of study time; no incidents were reported.

**Table 1:** Trial packs.

Trial packs	A	B	C
Combination	Diclofenac (IM)	Paracetamol (IV)	Paracetamol (oral)
1	Yes	Placebo	Yes
2	Yes	Yes	Placebo
3	Yes	Placebo	Placebo

### 2.4. Outcomes

The primary outcome measured was the difference in mean pain reduction at t30. Secondary outcomes included the difference in the proportion of patients who experienced at least 50% pain relief at t30, as well as the need for rescue analgesia and the rate of adverse events at t30.

### 2.5. Statistical Analysis

The sample size was determined based on the average pain reduction reported in prior studies with paracetamol and diclofenac. Midpoint of the mean reduction, along with the largest standard deviation, was 4.3 ± 2.3 for paracetamol and 5.9 ± 2.2 for diclofenac [20–23]. Several previous studies evaluating the minimal clinically significant difference in pain scores using various assessment tools have reported a broad range, from 13 mm on the visual analog scale (VAS), 1.3 on the NRS [24, 25], to a 2.0‐point decrease on the VNRS [26]. For this trial, a predetermined value of 2.0 was selected as the minimum clinically significant change in pain scores. Anticipating a dropout rate of 15%, an additional seven subjects per group were enrolled, resulting in 54 subjects per group and a total sample size of 162 participants.

The analysis and plotting were conducted using STATA software. Continuous and normally distributed data variables are presented as means (±SD), or as medians with 95% confidence intervals (CIs), and non‐normal data are reported as medians with interquartile ranges (IQRs). Categorical variables are expressed as proportions. Descriptive statistics for categorical data were calculated using proportions, and key proportions are reported with binomial exact 95% CIs. One‐way ANOVA was performed for pain scores at each time point. Simple linear regression was applied to pain scores at time points t15 and t30 to assess any association between the two variables. For categorical outcomes, the Pearson X2 test was used as the global test of significance, and the Kruskal–Wallis test was employed for nonparametric outcomes. Multivariate logistic regression was carried out using stepwise and purposeful selection processes [27]. The statistician was blinded to the group assignments during the analysis phase.

### 2.6. Role of Funding Sources

The funding body was not involved in the study design, data collection, analysis, or writing of the manuscript.

## 3. Results

**Figure 1:**
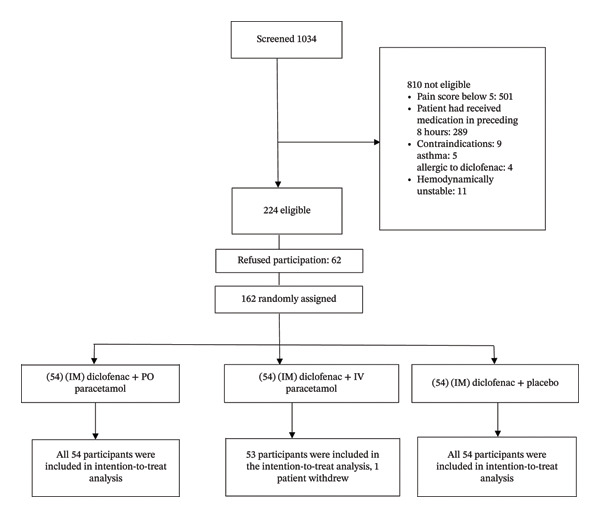
Trial profile.

The trial was conducted from October 15, 2022, through February 21, 2023. The research team screened 1034 patients, of whom 162 were enrolled in the study (see Figure 1).

All enrolled participants were randomized into one of three groups: IM diclofenac plus PO paracetamol (54), IM diclofenac plus IV paracetamol (53), or IM diclofenac only (54). One participant in the diclofenac plus IV paracetamol group withdrew and was not included in the analysis, as this individual did not complete drug administration; therefore, data from t15 to t90 were not recorded. The baseline characteristics of all three groups were similar (see Table 2).

**TABLE 2:** Patient characteristics.

Characteristic	D + POP: *n* = 54	D + IVP: *n* = 54	D + PLACEBO *n* = 54
Age (mean and standard deviation)	35.1 (±8.2)	34.0 (±8.3)	32.4 (±7.4)
*n* (%)	54 (100%)	54 (100%)	54 (100%)
Weight (Kg) (mean, SD)	70.7 (±10.5)	73.9 (±16.0)	69.6 (±12.1)
*n* (%) outdoor laborer occupation	40 (74%)	31 (57%)	32 (59%)
Nationality			
Africa	8 (14.8%)	0 (0.0%)	6 (11.1%)
Bangladesh	11 (20.3%)	10 (18.5%)	16 (29.6%)
India	18 (33.3%)	19 (35.1%)	13 (24.0%)
Nepal	10 (18.5%)	12 (22.2%)	10 (18.5%)
Pakistan	4 (7.4%)	8 (14.8%)	6 (11.1%)
Philippines	2 (3.7%)	1 (1.8%)	1 (1.8%)
Sri Lanka	1 (1.8%)	4 (7.4%)	2 (3.7%)
Injury characteristics			
*n* (%) upper (vs. lower) limb	28 (51.8%)	37 (68.5%)	34 (62.9%)
*n* (%) proximal (to wrist or ankle) vs. distal	45 (83.3%)	43 (79.6%)	42 (77.7%)
*n* (%) with blunt (vs. penetrating) injury	5 (9.2%)	9 (16.6%)	6 (11.1%)
*n* (%) diagnosed with fracture/dislocation	22 (40.7%)	27 (50.0%)	36 (66.6%)
Initial pain numeric rating score (NRS)			
t0	8.0 (±0.16)	7.7 (±0.16)	8.0 (±0.16)

All participants enrolled in the trial were male, as the study took place in a community general hospital that serves only adult male patients and is situated in the city’s industrial area. The initial mean pain score for all participants at time t0 was comparable, averaging 7.9 (SD ± 1.2).

### 3.1. Primary Outcome

Table 3 presents the average pain scores for participants across all three groups. At t15, none of the groups achieved the predefined clinically significant reduction of 2 points. However, by the primary outcome point at t30, the mean pain scores for all groups had decreased, meeting the threshold for clinical significance. Specifically, the mean pain scores at t30 were as follows: IM diclofenac plus PO paracetamol, 5.5 (95% CI, 5.1–5.9); IM diclofenac alone, 5.8 (95% CI, 5.4–6.3); and IM diclofenac plus IV paracetamol, 5.1 (95% CI, 4.7–5.5).

**Table 3:** Primary and secondary outcomes.

	**D + POP: *n* = 54**	**D + IVP: *n* = 53**	**D + PLACEBO *n* = 54**

Pain numeric rating score at time t15, t30, t45, t60, t75, t90 (mean, SD)			
t0	8.0 (±0.16)	7.7 (±0.16)	8.0 (±0.16)
t15	6.5 (±0.20)	6.1 (±0.16)	6.8 (±0.18)
t30	5.5 (±0.19)	5.1 (±0.19)	5.8 (±0.20)
t45	4.8 (±0.17)	4.2 (±0.21)	5.1 (±0.22)
t60	3.8 (±0.20)	3.4 (±0.22)	4.3 (±0.22)
t75	3.1 (±0.19)	2.5 (±0.24)	3.6 (±0.24)
t90	2.3 (±0.19)	1.9 (±0.22)	3 (±0.26)
Primary outcome (mean pain reduction) at t30	5.5 (95% CI 5.1—5.9)	5.1 (95% CI 4.7—5.5)	5.8 (95% CI 5.4—6.3)
Secondary outcomes			
Proportion of patients achieving 50% or more reduction in pain score at t30	4 (7.4%)	8 (15.0%)	6 (11.1%)
2‐point NRS drop within t90	(54) 100%	(52) 98%	(54) 100%

### 3.2. Secondary Outcomes

**Figure 2:**
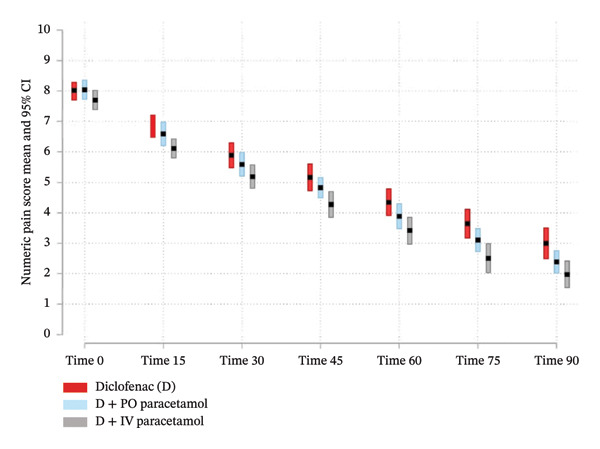
Mean (95% CI) pain scores for three study groups.

At the 30‐min mark (t30), 8 patients (15.0%) in the IM diclofenac plus IV paracetamol group achieved 50% pain relief, which was higher than the 6 patients (11.1%) in the IM diclofenac plus placebo group and the 4 patients (7.4%) in the IM diclofenac plus oral paracetamol group. No participants in any group required rescue analgesia at any time. At t30, all participants were asked by the treating physician if they needed rescue medication, and all declined. No adverse events were reported at or after t90 in any group. The IM diclofenac plus IV paracetamol group consistently reported lower pain scores at each time point; however, at no point did the reduction reach the predefined threshold of two points considered clinically significant (see Figure 2).

## 4. Discussion

In this study evaluating three analgesic regimens—IM diclofenac with either oral or IV paracetamol, or placebo—the average pain scores at 30 min (t30) did not differ significantly among the groups. Each group experienced a reduction in pain score that exceeded the predetermined threshold of 2 points on the NRS, indicating a clinically meaningful decrease in pain at the 30‐min mark [24, 25]. Across all time points measured, every combination offered comparable analgesic effects, with reductions in pain scores reaching the clinically significant level as specified beforehand, starting from the 15‐min time point (t15). The pairing of IV paracetamol with IM diclofenac resulted in marginally lower pain scores at each interval compared to the combination with oral paracetamol or placebo, but these differences were not clinically significant.

Most prior research on combination analgesia has focused more on oral agents than parenteral combinations. Earlier studies evaluating the efficacy of oral analgesic combinations, compared to single agents in the ED, generally found no statistically significant or clinically meaningful differences among commonly used analgesics, such as NSAIDs or paracetamol [16, 28–31]. A qualitative systematic review by Ong et al. on combining paracetamol with NSAIDs for acute postoperative pain reported that “overall, 85% of trials comparing paracetamol and NSAID versus paracetamol alone, and 67% of trials comparing paracetamol and a NSAID versus a NSAID alone, provided more effective pain relief of the combination, compared to single drug” [32]. Additionally, a Cochrane review of oral combinations of NSAIDs and paracetamol for dental procedures concluded that “combination therapy (both ibuprofen 200 mg/paracetamol 500 mg, and ibuprofen 400 mg/paracetamol 1000 mg) is more effective than placebo, or single dose of ibuprofen. The Number Needed to Treat (NNT) was 1.6, and NNT 1.5, respectively, concluding ibuprofen 400 mg/paracetamol 1000 mg is more effective than ibuprofen alone (NNT 5.4)” [33]. Furthermore, an animal study by Miranda et al. in 2006 examining the synergism between paracetamol and NSAIDs in experimental acute pain suggested potent interactions between these drugs and supported the clinical use of their combinations for treating pain conditions [34].

No substantial difference in pain scores was found between oral and IV administration of paracetamol at any measured interval. This finding challenges the commonly held belief that the parenteral route delivers a more rapid effect than oral medication [35]. The swift onset of action seen with IM diclofenac may account for this, as paracetamol did not provide any additional benefit. Previous research has indicated there is no clear advantage in analgesic effectiveness of IV paracetamol over the oral form [36, 37]. Furthermore, oral preparations are often preferred by patients and may even act faster, thereby negating any theoretical benefit of IV administration, as well as avoiding the considerable cost associated with IV paracetamol [38].

Additionally, the study found that the combination of diclofenac with IV paracetamol resulted in a greater proportion of patients experiencing at least a 50% reduction in pain at 30 min compared to the combination of oral paracetamol with IM diclofenac or IM diclofenac alone. Achieving a relative pain reduction of 50% or more, along with an absolute reduction of at least 2.5 cm on the VAS, is considered clinically meaningful in patients undergoing third molar surgery [39]. Enhanced early pain management may improve patient comfort and decrease length of stay [40]. In the ED, combining IM and oral medications may be faster to administer than combining IM and IV medications due to preparation time [41]. Although the statistical improvement among the three group combinations was only marginal, the adjunctive effect of IV paracetamol with IM diclofenac was slightly more notable than with oral paracetamol.

No adverse events or requirements for rescue analgesia were observed during the study, consistent with findings from previous research on paracetamol and NSAIDs [42]. This outcome may be attributed to the predominantly younger study population, who had few comorbidities. While there are potential concerns about delayed complications associated with NSAIDs—such as IM necrosis, abscess formation, bleeding, acute kidney injury, and, in rare instances, death—none of these issues were reported in this study.

## 5. Limitations

This study has several limitations, chief among them being its recruitment from a single center, which was a male‐only hospital. Nevertheless, the male study population represented a culturally diverse group. According to a 2015 study on gender differences in occupational injury incidence, physical injuries are 1.4 times more common among males than females [43]. Although the current trial is limited to the male population, musculoskeletal injuries contribute substantially to disability and suffering not only in high‐income countries, but even more so in low‐ and middle‐income countries [44].

Another limitation involves the monitoring period for adverse events, which was restricted to the 90‐min duration of the study and could potentially have led to an underreporting of such occurrences. After this timeframe, participants were returned to the ED physician, who continued to oversee their care until their final disposition. Notably, no adverse events were documented during the study, nor were any reported by the treating physicians afterward, as confirmed in their feedback.

Additionally, pain scores, while useful for assessing subjective severity, may not fully capture the complexity of the nature of underlying injury mechanisms. It is possible that significant differences exist in the type of injuries sustained by each group; however, they are not reflected by pain assessment alone. The mechanism of injury distribution encountered is diverse in this study and would not be representative of all clinical settings. Further stratification by injury mechanisms would have provided additional insight related to injury patterns and outcomes. Nevertheless, performing subgroup analysis with the current dataset is rendered statistically underpowered due to the limited number of cases. Recommendation for future studies with larger, and more diverse, cohorts, including careful clinical and data reporting, for more definitive conclusions about the impact of injury mechanism on outcomes.

## 6. Conclusion

Both IV and oral paracetamol, when combined with IM diclofenac or administered alone, provide comparable levels of pain relief in patients with acute limb injuries. Further studies exploring different NSAID and paracetamol combinations, evaluating a broader range of adverse events, and assessing the need for rescue analgesia are recommended.

## Author Contributions

Isma Qureshi, MBBS, PGDCC, Clinical Research Specialist: study concept including protocol writing, design and methodology, project administration, logistics, and manuscript writing. The author is familiar with the content of the manuscript and is included in the final approval of the manuscript.

Raheel S. Qureshi, MD, Consultant Emergency Department: study concepts including protocol writing, design and methodology, project administration including logistics and data collection. The author is familiar with the content of the manuscript and is included in the final approval of the manuscript.

Tim Harris, MD, FACEM., FRCEM, FFICM, Professor Emergency Medicine: study concept including revising protocol, design and methodology, and manuscript revision. The author is familiar with the content of the manuscript and is included in the final approval of the manuscript.

Sameer A. Pathan, MD, FRCEM, PhD, Consultant Emergency Department: study concepts including revising protocol, design and methodology, and manuscript review. The author is familiar with the content of the manuscript and is included in the final approval of the manuscript.

Ashraf M. El Malik, MD, Clinical Pharmacist: study concept including protocol writing, design and methodology, and pharmacy supervision related to drug stability and blinding process. The author is familiar with the content of the manuscript and is included in the final approval of the manuscript.

Mohd E. Babiker, MD, Pharmacy Supervisor, pharmacy supervision helping with trial packet preparation. The author is familiar with the content of the manuscript and is included in the final approval of the manuscript.

Mohamad A. Jamali MD, Staff Pharmacist: Trial packet preparation and dispensing during enrollment. The author is familiar with the content of the manuscript and is included in the final approval of the manuscript.

Zain A. Bhutta, MD, Clinical Research Specialist: study concept and protocol writing, design and methodology, and manuscript writing. The author is familiar with the content of the manuscript and is included in the final approval of the manuscript.

Stephen H. Thomas, MD, MPH, Professor Emergency Medicine: study concepts and protocol reviewing, design and methodology, and statistical analysis for the results. The author is familiar with the content of the manuscript and is included in the final approval of the manuscript.

## Funding

The trial was funded by Medical Research Center, Hamad Medical Corporation, Qatar (Approval number # IRGC‐04‐NI‐17‐099).

## Ethics Statement

The trial was approved by the Ethics Committee of the Institutional Review Board of Medical Research Center, Hamad Medical Corporation, Qatar (Approval number # IRGC‐04‐NI‐17‐099), on November 27, 2018.

## Consent

Informed consent was obtained from all subjects and/or their legal guardian(s).

## Conflicts of Interest

The authors declare no conflicts of interest.

## Data Availability

The data that support the findings of this study are available upon request from the corresponding author (iqureshi@hamad.qa). The data are not publicly available due to privacy or ethical restrictions.
